# 92741 Racial differences in patient-reported distress among women with endometrial cancer

**DOI:** 10.1017/cts.2021.625

**Published:** 2021-03-31

**Authors:** Hadley Reid, Mary Katherine Montes de Oca, Oluwadamilola M. Fayanju, Laura J. Havrilesky, Brittany A. Davidson

**Affiliations:** Duke University Medical Center

## Abstract

ABSTRACT IMPACT: This work will inform and improve the way we assess and treat distress in women with endometrial cancer. OBJECTIVES/GOALS: Distress from cancer is associated with worse processes of care. Differences in outcomes by race/ethnicity in endometrial cancer (EC) are well documented, but differences in distress have not been previously explored. Here we characterize the association between race/ethnicity, distress scores, and stressors reported by patients with EC. METHODS/STUDY POPULATION: Patients presenting to a single academic outpatient gynecologic oncology practice for initial evaluation of known EC from January 2013-May 2020 were included. The electronic health record was used to abstract demographics, National Comprehensive Cancer Network Distress Thermometer and Problem List (NCCN DT) scores and stressor categories (physical, emotional, spiritual, practical, and family) from the initial encounter. Referral to support services occurs at NCCN DT score ≥4. We excluded women who received prior cancer-directed therapy and those without an initial NCCN DT score. Summary statistics were tabulated for demographics. Mann-Whitney U tests were used for inter-group difference on continuous variables and 2-sample tests for equality of proportions were used for binary variables. RESULTS/ANTICIPATED RESULTS: 412 non-Hispanic White (NHW, mean age 63) and 149 non-Hispanic Black (NHB, mean age 65) women were included in our analysis. More NHB women presented with high-grade EC (53.7%) vs NHW women (21.9%) and fewer NHB women were privately insured (32% vs 52%). Median distress scores were higher in NHW women compared to their NHB counterparts (4 vs. 2, p<0.001) and NHB women were more likely to report a distress score of 0 compared to their NHW counterparts (32% vs 19%, p=0.001). 50.5% NHW women had a score ≥4 and thus qualified for referral to services compared to 20.7% of NHB women (p=0.02). Of those referred, NHB and NHW women declined referral to support services at similar rates (35.1% vs 34.5%; NS). There was a significant difference in the median number of stressors reported by NHW and NHB women, (4 vs 3 stressors; p=0.02). DISCUSSION/SIGNIFICANCE OF FINDINGS: The NCCN DT, a widely used tool in cancer clinics, may fail to adequately measure distress in NHB women presenting with a diagnosis of EC, despite >30% more high-risk histology cancers in this cohort. This difference leads to disparities in referral to additional support services, which may affect quality of care and quality of life.

